# Supplementary material for: Bioavailability Assessment of Yarrow Phenolic Compounds Using an In Vitro Digestion/Caco-2 Cell Model: Anti-Inflammatory Activity of Basolateral Fraction
Source: Molecules. 2022 Nov 26;27(23):8254. doi: 10.3390/molecules27238254 (PMC9740014; doi:10.3390/molecules27238254)
Supplement: Supplementary file 1 [file molecules-27-08254-s001.zip › molecules-2000295-supplementary.pdf]

Supplementary material

Bioavailability assessment of yarrow phenolic compounds using an in vitro digestion/ Caco-2 cell model: anti-inflammatory activity of basolateral fraction.

Marisol Villalva[1], Laura Jaime[1], María de las Nieves Siles-Sánchez[1], Susana Santoyo[1*]

[1] Institute of Food Science Research (CIAL). Universidad Autónoma de Madrid (CEI UAM+CSIC). 28049 Madrid, Spain

* Correspondence: susana.santoyo@uam.es (S.S.), Tel.: +34 91 001 79 26

Table S1. Phenolic compounds identified in yarrow samples by using HPLC-PAD-ESI-QTOF-MS/MS in negative ionization mode.

No.	Rt	Compound	Acc. Mass (*m*/*z*)	Theor. Mass (*m*/*z*)	Error (ppm)	Main fragments (*m*/*z*)
1	12.90	Neochlorogenic acid *	353.0877	353.0878	0.2	191.0561, 179.0379, 135.0454
2	13.35	Protocatechuic acid *	153.0197	153.0193	-2.5	109.0299, 153.0197
3	13.62	Caftaric acid isomer	311.0405	311.0409	1.0	179.0354, 149.0458
4	13.72	Caftaric acid *	311.0410	311.0409	-0.3	179.0353, 149.0460
5	14.36	Caffeoylquinic acid isomer I	353.0876	353.0878	0.5	191.0561, 179.0350
6	14.94	Chlorogenic acid *	353.0876	353.0878	0.7	191.0559
7	15.15	Cryptochlorogenic acid *	353.0876	353.0878	0.6	173.0455, 179.0349
8	15.93	Vicenin 2 *	593.1513	593.1512	-0.2	473.1093
9	17.02	Caffeoylquinic acid isomer II	353.0879	353.0878	-0.2	191.0565, 161.0248
10	17.45	Apigenin hexoside-pentoside I	563.1401	563.1406	1.0	473.1089, 443.0979
11	17.88	Caffeic acid *	179.0349	179.0350	0.4	135.0451
12	18.12	Schaftoside isomer	563.1401	563.1406	1.0	473.1089, 443.0979
13	18.26	Schaftoside *	563.1406	563.1406	0.1	473.1090, 443.0984
14	18.85	Homoorientin *	447.0933	447.0933	-0.0	357.0614, 327.0509, 429.0826
15	19.32	Apigenin hexoside-pentoside II	563.1408	563.1406	-0.3	443.0983, 473.1086
16	19.58	Luteolin dihexoside I	609.1462	609.1461	-0.1	447.0931, 327.0509, 357.0615, 285.0405
17	19.90	6-hydroxyluteolin-7-*O*-glucoside	463.0885	463.0882	-0.7	301.0351
18	20.18	Apigenin dihexoside	593.1511	593.1512	0.2	269.0453
19	21.15	Quercetin hexoside	463.0883	463.0882	-0.2	301.0354
20	21.60	Luteolin dihexoside II	609.1449	609.1461	1.9	285.0406, 447.0930
21	21.92	Rutin *	609.1454	609.1461	1.2	301.0351
22	22.28	Apigenin hexoside	431.0983	431.0984	0.2	311.0559
23	22.44	Vitexin *	431.0981	431.0984	0.6	311.0562, 341.0666
24	22.90	Apigenin hexoside-deoxyhexoside	577.1557	577.1563	1.0	269.0455
25	23.47	Apigenin derivative	445.1141	445.1140	-0.1	269.0450
26	23.64	Luteolin-7-*O*-glucoside *	447.0934	447.0933	-0.2	285.0403
27	24.11	Luteolin-7-*O*-glucuronide *	461.0729	461.0725	-0.8	285.0404
28	24.67	Quercetin hexuronide	477.0678	477.0675	-0.7	301.0352
29	25.47	3,4-Dicaffeoylquinic acid *	515.1195	515.1195	-0.0	353.0878, 335.0773, 191.0561, 179.0350
30	25.93	Isorhamnetin hexoside I	477.1035	477.1038	0.8	315.0721, 300.0270
31	26.33	1,5-Dicaffeoylquinic acid *	515.1195	515.1195	-0.0	353.0874, 191.0559, 179.0349, 135.0453
32	26.88	3,5-Dicaffeoylquinic acid *	515.1194	515.1195	0.1	353.0872, 191.0561, 179.0351, 135.0455
33	27.60	Apigenin-7-*O*-glucoside *	431.0983	431.0984	0.2	269.0449
34	27.86	Luteolin-*O*-malonylglucoside	533.0939	533.0937	-0.4	489.1037, 285.0403
35	28.25	4,5-Dicaffeoylquinic acid *	515.1196	515.1195	-0.1	353.0877, 191.0561, 179.0350
36	30.37	Isorhamnetin hexoside II	477.1042	477.1038	-0.6	315.0720
37	31.09	Dicaffeoylquinic acid isomer	515.1191	515.1195	0.7	353.0878, 191.0565, 179.0353, 173.0459
38	32.04	Feruloylcaffeoylquinic acid	529.1350	529.1351	0.1	367.1036, 193.0510, 191.0565
39	32.70	Tricaffeoylquinic acid	677.1514	677.1512	1.9	515.1197, 353.0880
40	33.61	Luteolin *	285.0401	285.0405	1.4	151.0039, 133.0298
41	33.80	Quercetin *	301.0351	301.0354	0.8	151.0037, 121.0296, 107.0141
42	35.04	Methoxyquercetin	315.0507	315.0510	0.6	300.0272, 271.0245, 255.0296, 301.0307
43	36.99	Apigenin *	269.0454	269.0455	0.4	151.0039, 225.0558
44	37.51	Diosmetin *	299.0554	299.0561	2.4	284.0325, 285.0383
45	38.49	Trihydroxy dimethoxyflavone	329.0665	329.0667	0.5	299.0195, 314.0426
46	40.00	Centaureidin	359.0768	359.0772	1.0	329.0299, 344.0535, 314.0066
47	42.04	Methoxyacacetin	313.0715	313.0718	0.9	283.0245, 298.0476, 284.0278
48	43.12	Dihydroxy trimethoxyflavone	343.0821	343.0823	0.7	328.0585, 313.0350
49	45.51	Casticin *	373.0925	373.0929	0.9	343.0457, 358.0691

Rt, retention time. * Comparison against its authentic standard

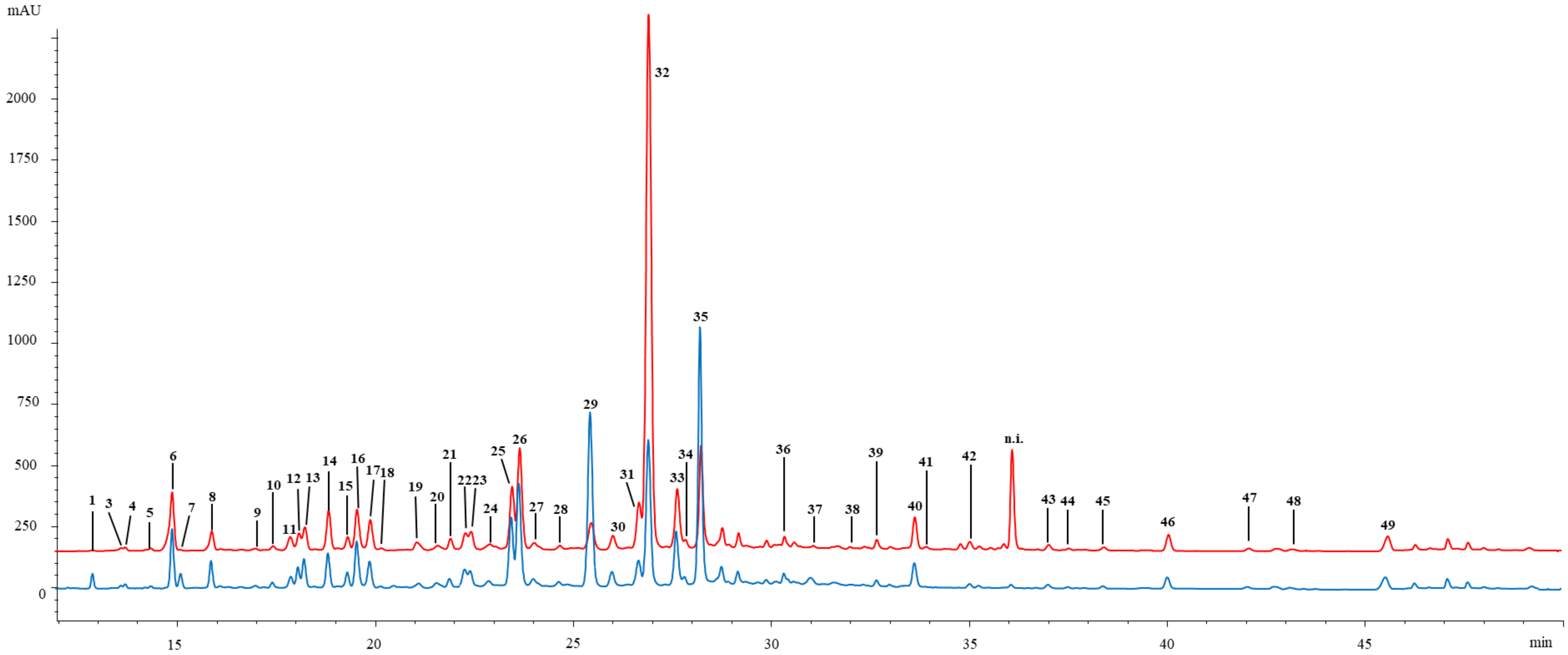

Figure S1. HPLC-PAD base peak chromatogram (λ= 320 nm) of yarrow enriched-extract before (red line) and after (blue line) the simulated *in vitro* digestion process. n.i.: non-identified compound.
